# Exploring Recent Trends in Cervical Cancer Screening Uptake in Tanzania

**DOI:** 10.24248/eahrj.v9i2.857

**Published:** 2025-12-24

**Authors:** Joanes Faustine Mboineki, Changying Chen professor

**Affiliations:** a Department of Nursing Management and Education, School of Nursing and Public Health, The University of Dodoma, Dodoma, Tanzania; b The First Affiliated Hospital of Zhengzhou University, Zhengzhou, China; c School of Nursing and Health of Zhengzhou University, Zhengzhou, China

## Abstract

**Background:**

Despite ongoing community awareness programmes in Tanzania, cervical cancer screening (CCS) uptake remains low, with only 10 to 29% of eligible women participating. Improving screening is essential to achieving Sustainable Development Goal 3, which targets reducing premature mortality from non-communicable diseases. This study explored recent changes in CCS awareness, knowledge, attitudes, and practices among women in urban Dar es Salaam.

**Methods:**

A qualitative descriptive study was conducted in Kawe ward, Dar es Salaam. Twenty-six women aged ≥21 years were purposively sampled. Data were collected between January and February 2020 using in-depth, face-to-face interviews guided by open-ended questions. Interviews were audio-recorded, transcribed verbatim, translated, and analysed using five-step qualitative content analysis to develop codes, categories, subthemes, and themes.

**Results:**

All participants had heard of cervical cancer and CCS, yet only 2 of 26 had ever been screened. Awareness was high, but most women demonstrated inadequate knowledge of cervical cancer causes, risk factors, symptoms, prevention, and screening guidelines. Major barriers to screening included absence of symptoms, limited knowledge, lack of health-seeking behaviour, time constraints, perceived costs, and long waiting times at screening sites. Despite these barriers, participants expressed strong positive attitudes toward CCS and showed readiness to undergo screening if services were accessible, affordable, and accompanied by adequate education.

**Conclusion:**

While awareness of cervical cancer is high, knowledge gaps persist and remain a major barrier to CCS uptake. Ongoing community awareness initiatives have increased recognition of the disease's severity but have not translated into improved screening practices. Strengthening educational content, expanding community-level outreach, reducing waiting times, and improving service accessibility are essential to increase CCS participation among urban women in Tanzania.

## BACKGROUND

Even though the era of Sustainable Development Goal 3 aims to reduce one-third of premature mortality from non-communicable diseases by 2030 through prevention and treatment,^1^ cervical cancer remains the fourth most common cancer among women worldwide, and in 2020, an estimated 604,000 new cases were diagnosed, and 342,000 among them died worldwide.^2^ Developing countries account for 85% of all cervical cancer deaths worldwide, and over 90% of the highest incidence rates of cervical cancer occur in sub-Saharan Africa.^3^ Despite the fact that screening is the primary prevention and intervention measure to reduce the incidence and mortality rate of cervical cancer, ^4^ the delivery and utilization of screening services remain a critical challenge in low-resource settings.^5^

Cervical cancer in Tanzania remains the most common cancer for women, with an estimated agestandardized incidence rate of 54 per 100,000 women per year,^6^ which is 3.9 times higher than the global disease incidence rate.^7^ Cervical cancer in Tanzania accounts for 6695 deaths each year,^8^ with a projection of 9,923 deaths per year in 2025.^9^

Regarding cervical cancer services in Tanzania, the specialized cancer services are provided by Ocean Road Cancer Institute (ORCI) only.^7^ The institute deals with treatment (radiotherapy & chemotherapy), early detection, teaching, and prevention. Currently, ORCI has an inpatient capacity of not less than 270 beds, while the number of outpatients attended per day is estimated to be about 250 patients on a heavy clinic day and about 200 patients on a light clinic day.^10^ The screening program was introduced in Tanzania in 2011.^11^ The National cervical cancer screening programme in Tanzania adopted the Visual Inspection with Acetic acid (VIA) test as an alternative screening method to the Papanicolaou (Pap) test, which is available free of charge to any eligible woman, aged 18 to 55 years.^12,13^

Although the government has established numerous screening facilities across the country and trained oncology experts to increase the accessibility of screening services, there is poor participation in the cervical cancer screening tests,^14–16^ with an estimated 10%–29% of the population participating.^17^ This leads to 75%-80% of women attending the hospital at an advanced stage when the disease is impossible to cure.^9^ Dar es Salaam is a business city with a female population of 2.2 million, and with a specialized cancer hospital named Ocean Road Cancer Institute (ORCI) located.^9^ Most of the government efforts in preventing cervical cancer were implemented in this region, yet a small number of women, at 4%-14.7% from this region, participate in CCS.^18,19^

The screening barriers among women in rural and urban areas are similar.^6^ Many barriers to cervical cancer screening have been previously reported in different studies, but a knowledge deficit about the disease remains a predominant factor.^20^ The cervical cancer screening programme in Tanzania established a community awareness education program, which is still ongoing in different parts of the country. By definition, a community awareness education program refers to a structured program launched in 2017, made up of oncology experts, whose role is to inform communities about cervical cancer screening and purposely promote awareness. Awareness in this study is defined as women being heard about cervical cancer and CCS, while knowledge level refers to the ability of participants to provide a comprehensive explanation about cervical cancer, causes, risk factors, signs and symptoms, prevention, screening tests and screening guidelines. The impact of the community awareness education program was evaluated by Kilimanjaro Christian Medical Center (KCMC), which reported that 81% of women were aware of cervical cancer screening, but only 22% were knowledgeable about cervical cancer screening.^21^ Since the community education programs are in progress, there might be some further changes in awareness, knowledge and CCS among women. However, this study hypothesizes that regardless of the ongoing community awareness education programs in the urban area of Dar es Salaam, the screening status and knowledge level remain unchanged.

## METHODS

### Study Design

The study adopted a qualitative descriptive design to explore changes in cervical cancer screening among women in urban Dar es Salaam.

### Setting and Sample

The study was conducted in the city of Dar es Salaam, in Kawe ward in Tanzania. The ward has four neighborhoods, whereby Mzimuni was selected for the study due to the availability of a cervical cancer screening facility. The inclusion criteria were women aged 21 years and above based on eligibility recommendation,^22^ and able to communicate in Swahili's native language fluently. The study excluded women who presented any sign of mental illness and those unwilling to participate in the study. A community-based purposive sampling was applied to obtain the participants. The sample size of twenty-six was considered adequate when the saturation point was reached. The saturation was considered when participants provided no new ideas or opinions during the interview.^23^

### Data Collection Procedures

The study was conducted from 21st January 2020 to 04th February 2020. An in-depth interview approach was used with Key Informants to explore trends in cervical cancer screening among twenty-six participants. Purposive sampling was applied to obtain participants from households, and In-depth, face-to-face interviews were conducted using an interview guide with open-ended questions.

The interview guide was developed based on a literature review and experts’ evaluation. 24 The final version of the interview guide had two main questions and four probes as follows: MAIN QUESTION 1: What do you understand about cervical cancer and cervical cancer screening? PROBE 1: What does it mean by cervical cancer? PROBE 2: What is cervical cancer screening? MAIN QUESTION 2: How are your practices on cervical cancer screening? PROBE 1: How often do you participate in cervical cancer screening? PROBE 2: What are the reasons hindering you from taking cervical cancer screening? Other probing questions that were often asked are: “Can you explain more about what you have just said? “Can you give an example of what you have mentioned?” “Do you think there are still other issues you want to speak about?” “Has anything important been left out or forgotten that you would like to share?” During the interview, an audio recorder was used to keep the interview discussions for future analysis. The sample size was considered adequate after the saturation level was achieved. It was when the participants repeated similar information to the previous participants, and no new information came out, that a saturation point was considered.

### Ethics Approval and Consent to Participate

The study was ethically approved by the University of Dodoma Ethical Research Committee, with identification number UDOM/DRP/134/VOL IV/42. The committee confirmed that the study contained no form of harm to participants. Written informed consent was obtained from each participant before participating in the study. The confidentiality of participants’ information was maintained through avoiding transcripts and audio recordings accessible to non-researchers. During the interview, participants were reminded to avoid revealing their identification, such as their real names, their residential areas, or their leaders. Participants were free to participate or withdraw from the study at any time they felt to do so.

### Data Analysis

Data were transcribed verbatim and translated from the native Swahili language into the English language by the principal investigator with his research assistant, who are fluent in both languages. Two individuals coded the data by reading and re-reading the transcripts to identify emerging concepts. In the review, the phrases which captured the emerging concepts were coded and analyzed to determine the similarities and differences.^25^ Content was arranged logically to identify the focus, and qualitative content analysis was used to identify explanatory themes based on research objectives. Since content analysis is categorized as conceptual analysis and relational analysis, the current study applied relational analysis, whereby the concepts were chosen for examination and exploration of the relationships between them.^26^ The study adopted a five-step qualitative content analysis: (i) Meaning unit, (ii) code, (iii) categories, (iv) subthemes, and (v) themes.^27^

## RESULTS

### Demographic Characteristics

The mean age of participants was 34.73 years, with 15 (57.7%) in the age group of 30–65 years and 11 (42.3%) in the age group of 21–29 years. The participants 22 (84.6%) were employed or self-employed, and among them had a primary school level of education 14 (53.8%). Meanwhile, 22 (84.6%) were Christian, and 4 (15.4%) were Muslim. Among them, 16 (61.5%) were single and 10 (38.5%) were married women. It was found that 24 (92.3%) participants had never screened for cervical cancer. The mean total pregnancies among participants was 1.42 children. The average duration of the interview was 5.8 minutes. Refer to the summarized information in Table 1.

**TABLE 1:** Demographic Characteristics

Variables	Frequency (N=25)	Proportion (%)
Age (Mean=34.73) 22 ± 57		
30–65 years	15	57.7
21–29 years	11	42.3
Occupation		
Employed	22	84.6
Housewife	3	11.5
Not employed	1	3.8
Educational level		
College/University level	3	11.5
Advanced level of education	1	3.8
Ordinary level of education	7	26.9
Primary school level of education	14	53.8
Unfinished primary school	1	3.8
Religion		
Christian	22	84.6
Muslim	4	15.4
Marital status		
Married	10	38.5
Single	16	61.5
Ever screened for cervical cancer		
Yes	2	7.7
No	24	92.3

Total pregnancies (Mean=1.42) 0 ± 5

Interview duration (Mean=5.8 Minutes) 2.59 ± 11.20

### Themes

Seven main themes and twenty-one subthemes emerged in this study. The main themes were: awareness about cervical cancer and CCS, knowledge about cervical cancer and CCS, hindrances to undergoing CCS, perception toward CCS, influences to undergo CCS, readiness for CCS, and CCS practices. Refer to the summarized information from Table 2.

**TABLE 2:** Themes, Subthemes Emerged in the Study

Themes	Subthemes	Outcome
1. Awareness about cervical cancer & CCS	Source of information Attentive to the information	Women had higher level of awareness on CC and CCS.
2. Knowledge about cervical cancer & CCS	Locate the area where CC occurs Understanding the basic knowledge of CC; causes, risks, signs and symptoms, prevention & CCS test & guidelines	Respondents demonstrated inadequate knowledge regarding CC and CCS.
3. Hindrances to undergo cervical cancer screening	Being asymptomatic lack of community education programs Having no free time to attend screening services screening costs Have no reason	Inadequate knowledge about CC & CCS emerged as the common barrier in the participation of CCS
4. Perception toward cervical cancer screening	Attitude Recommended supporting environment to influence women to go for screening	Majority of women had positive attitude towards CCS
5. Influences to undergo cervical cancer screening	Being symptomatic Community sensitization Desire to know health status Availability of screening services Free time Free screening costs Personal decision	The delivery of community education regarding CC and CCS was found the prominent influence.
6. Readiness for cervical cancer screening	Readiness demonstration Suggested factors that would totally result in the participation of CCS	Majority of women were ready for screening only when some of factors are considered
7. Cervical cancer screening practices	Experiences in the participation of previous screening test (s)	The practice in the participation of CCS was not good as participants were not adherent to screening guidelines.

### Awareness about Cervical Cancer

All women in the present study self-reported having heard about cervical cancer and cervical cancer screening. However, fifteen among them reported knowing nothing about cervical cancer and CCS.

*“I have heard about cervical cancer screening, but I don't know the details about the disease (P, 1)”.*

*“I have heard many times the information regarding cervical cancer, but I know nothing about it (P, 18)”*

Fifteen respondents identified radios and televisions as their main sources of information about CC and CCS, while other women reported other sources.

*“I have heard about cervical cancer from the community awareness education program (P, 12)”*

*“I have never heard the information about cervical cancer from some advertisements (P, 2)”*

*“I have heard about cervical cancer from Social Media groups and from people around whom their friends/relatives/community people have died of this disease (P, 8)”.*

*“I have heard about cervical cancer through in clinic (P, 21)”*

Three participants who had heard about cervical cancer and CCS through awareness education programs and the Media reported paying no attention to understanding it.

*“I always hear on television concerning cervical cancer, but I don't become attentive to understand the information deeply (P, 11)”*

*“I have heard about cervical cancer on the radio, but I don't concentrate on the details provided (P, 26)”*

### Knowledge about cervical cancer

Sixth participants reported correctly that cervical cancer occurs in the cervix, which is part of women's reproductive organs.

*“It is cancer that invades the cervix, and the cervix is located internally in women's reproductive organs (P, 6)”*

*“I know it is the disease that affects the reproductive organs of women (P, 24)”*

It was found that nine participants demonstrated that cervical cancer is a dangerous disease, affecting the women's reproductive organs by causing barrenness, and any delay in treatment may lead to death.

*“Cervical cancer is very dangerous, and if there is any delay, it becomes a fatal problem (P, 13)”*

*“I know that when someone gets cervical cancer cannot bear a child (P, 22)”.*

*“Many women are dying from cervical cancer (P, 8)”*

Five participants who defined what cervical cancer is failed to locate the exact body part in women's reproductive organs where cervical cancer occurs. This woman reported that;

*“Cervical cancer can be in any part of the body (P, 4)” “I know that cervical cancer affects women's reproductive organs, but I don't know the exact part (P, 8)”*

*“I can't locate, but I know that the disease occurs in the uterus (P, 9)”*

*“I know that the disease occurs in the uterus, but I don't know the exact area where it occurs (P, 13)”*

Twenty participants demonstrated inadequate basic knowledge as they reported knowing nothing about cervical cancer definition, risk factors, cause, prevention, treatment, cervical cancer screening guidelines, and the VIA test.

*“I don't know about cervical cancer. I do not understand the cause, signs and symptoms, and prevention methods. Even I know nothing concerning cervical cancer screening, how the screening tests are performed, and where the screening service can be found (P, 2)”*

*“I know nothing about cervical cancer and cervical cancer screening, unless I got sick from this disease, I can understand it better (P, 17)”*

*“Although I was previously screened once for cervical cancer, I know nothing about cervical cancer causes, signs and symptoms, prevention, and cervical cancer screening. I just went for a screening to know my health status (P, 12)”.*

Two participants reported that they had never received any education about cervical cancer and cervical cancer screening, which is one reason influencing their inadequate knowledge.

*“I do not know anything about cervical cancer and CCS because I have never received education about it (P, 18)”*

Most women who responded to the cause of cervical cancer only demonstrated that this disease is caused when women use chemical products in their daily lives.

*“There are so many causes for cervical cancer, such as food and drinks (beverages) that are made in factories, which contain some chemicals that can lead to cervical cancer (P, 1)”*

*“If women during their bath use chemical products such as soap can lead to cervical cancer (P, 3)”*

*“Foods and drinks like soda can cause cervical cancer (P, 4)”*

Some other women self-reported more causes of cervical cancer;

*“I know the abortion can lead to cervical cancer (P, 6)”*

*“The cause of cervical cancer is when a woman has missed her menstruation for 2 months, which can lead to cervical cancer (P, 11)”*

Only one participant reported that cervical cancer is asymptomatic in the early stages, but the signs and symptoms become apparent as the disease advances.

*“I understand that you may have cervical cancer, and yet you do not know if you have it (P,11)”*

Five participants mentioned lower abdominal pain, abnormal vaginal discharge, and vaginal bleeding as signs and symptoms of cervical cancer.

*“Lower abdominal pain and abnormal vaginal discharges are signs and symptoms of cervical cancer (P,1)”*

*“I know that people with cervical cancer will have stomach pain and vaginal bleeding (P, 3)”*

A few participants self-reported other signs and symptoms that can be presented by patients with cervical cancer.

*“The sign of cervical cancer is pain during sexual intercourse and vaginal bleeding (P, 20)”*

*“Pain during menstruation is the symptom of cervical cancer (P, 9)”*

*“When a woman has reached menopause, but she has not removed the uterus, it can lead to cervical cancer (P, 8)”*

Women indicated a lack of knowledge about the clear meaning of the prevention of cervical cancer. They did not realize that women should go for screening even when they are asymptomatic; instead, they reported that prevention is when a woman immediately sees in herself some signs and symptoms of cervical cancer, and without delay, seeks screening services.

*“Women must seek screening once they feel something is not good in their body. This will help the early detection, early treatment, and cost-effectiveness in the treatment (P, 1)”*

*“When you feel any problem in your body and you go for screening, the surgery will be performed to remove the tumor (P, 4)”.*

Few participants were able to recognize the prevention measures that may help to reduce the incidence and prevalence of cervical cancer.

*“Avoiding multiple sexual partners and sexual intercourse at a young age can prevent CC (P, 5)”*

*“The prevention of cervical cancer should be promoted by community awareness education programs about the importance of screening; without this, many people, even when they see the signs and symptoms, become careless until the disease reaches an advanced stage (P, 1)”*

*“The prevention is by regularly checking the health status (P, 13)”.*

Some other women identified the prevention measures, but were not sure of their responses

*“Women should prevent themselves from cervical cancer by avoiding bathing with chemical products like soaps (P, 3)”*

*“The prevention should be avoiding eating processed food like (meat, beans) that stays for a long time after being manufactured (P, 4)”*

*“Women should prevent themselves from cervical cancer by using condoms during sexual intercourse, not abort the pregnancy, and seek medical intervention after seeing the signs and symptoms (P, 6)”*

*“The prevention of cervical cancer is to be clean in our private parts and choose good pads. For women using pieces of clothes during menstruation should ensure that those pieces are clean and ironed (P, 9)”.*

Only 9 participants discussed cervical cancer screening tests and guidelines. Among them, four participants had no idea about the CCS test and screening guidelines. Three participants they just knew screening tests as a means of detecting the existence of CC with no more information.

*“I don't know anything about cervical cancer screening, that's why I insist the community education should be provided to promote a clear understanding of the disease (P, 1)”*

*“I know the cervical cancer screening test as the test that detects the disease so that you early start the medical intervention (P, 13)”*

Only one participant who had a previous screening test was able to explain correctly how the screening test is being carried out.

*“I know the cervical cancer screening test, the doctors insert the device in the vagina to open it and observe if there is cancer or not (P, 6)”*

The majority of participants were not familiar with screening guidelines, especially those who must screen for cervical cancer. Out of twenty-four participants who responded to screening guidelines, five participants had no idea about the eligibility of women to undergo CCS, while fifteen participants reported that every woman aged 18 years old and above should undergo screening tests. Remained participants reported the following;

*“Any woman who has signs or symptoms of cervical cancer, especially body pain or other abnormal feelings, is eligible for screening tests (P, 1)”*

*“All women who have given birth or not yet should go for screening (P, 12)”*

*“Every woman is eligible to screen for cervical cancer, especially those from 16 years old and above (P, 13)”.*

*“Any woman should be screened for cervical cancer, especially those aged 35 years old and above (P, 19)”*

*“Women who have started giving birth are eligible for screening (P, 23)”.*

*“Any woman can undergo a screening test as long as she is of puberty age and above (P, 25)”.*

### Hindrances to undergoing cervical cancer screening

Women self-reported several reasons why they have not gone for screening tests. Out of twenty-four participants, nine among them felt not sick, which is why they did not decide to participate in CCS, and others had no health-seeking behavior for other diseases/conditions.

*“I have not gone for CCS because I'm not sick. When I experience some problem in my body, I will decide to undergo a screening test (P, 4)”.*

*“I have not screened because I don't have pain in my body (P, 21)”*

Four participants reported a lack of community education programs that would have imparted to them the knowledge of cervical cancer and influenced them to participate in CCS;

*“I have no education about cervical cancer, I don't know the causes of cervical cancer, that's why I have not decided to screen for cervical cancer (P, 26)”.*

*“I have not gone for screening because no one has encouraged or emphasized me to go for screening. If I were encouraged, I would go for screening (P, 2)”.*

The other four participants reported having no free time to attend screening services because of their busy schedules.

*“I have two children, I'm always busy taking care of them, I don't have free time to attend the screening test (P, 7)”.*

*“Last year, some healthcare professionals came to our street and invited women to go for screening, but on the days scheduled for screening, I had personal affairs that required me to travel away, this made me miss the opportunity (P, 3)”*

Two participants reported the screening costs as a hindrance to undergoing CCS, as revealed by some women.

*“I don't have money to attend the screening, as you know that nowadays without money you cannot do anything (P, 16)”.*

It was self-reported by four participants that they have no reason why they do not go for cervical cancer screening, but it is just a personal decision.

*“I don't have any reason why I have not screened for cervical cancer. It is just a decision, I have not yet decided, when I decide, I will go to screen (P, 18)”.*

*“I have not yet decided to go for a screening test (P, 20)”*

More reasons were reported by women that restrain them from attending and undergoing cervical cancer screening.

*“When they announced the availability of screening service at free costs, I have been trying to attend the screening, but I found many women in a long queue waiting for the service; therefore, I could not wait for the service (P, 8)”.*

*“I have not yet made a decision, although I have a habit of regularly checking my health status for simple tests, I don't go for big tests (P, 11)”.*

*“I have not gone for screening because I have not given birth to a child. Those who screen are supposed to have started giving birth (P, 24)”.*

*“I'm careless and procrastinating. I plan to go tomorrow, another tomorrow and finally I found myself unable to go for the screening (P, 13)”.*

### Attitude toward cervical cancer screening

All twenty-four participants demonstrated a positive attitude and readiness to undergo cervical cancer screening tests;

*“It is a good test; women should be screened for CC because this disease kills most women, and the disease does not have a treatment. After you get the tumor, it grows and finally you die with it (P, 4)”.*

*“It is a good test; I wish it could be offered regularly or even once per year. Unfortunately, this offer of the test is rarely provided (P, 3)”*

Some women emphasized that, though the test is good, there should be a supporting environment to influence women to go for screening.

*“If the screening service is available, free of charge and without follen (a long waiting time for services), it is very important for women to attend (P, 8)”*

*“Since the disease has a great effect on our society, this screening is very important. Much of awareness raising in communities should be performed, especially on causes, signs, symptoms, and prevention (P, 5)”.*

One participant disclosed having no perception of cervical cancer screening tests since she had never been educated.

*“I have no perception about cervical cancer screening because nobody has told me about it. If someone emphasizes me by helping me to know the cause, signs, and symptoms, treatments, etc, I will be ready to go (P, 2)”.*

### Influences to undergo cervical cancer screening

Women elaborated on what would make them able to attend the screening services. The five participants reported that when they get sick, they would go for screening tests.

*“It is until I'm sick or I see the signs and symptoms, I will decide to seek the screening services (P, 1)”.*

*“We have a habit of seeking medical attention once we get sick, but when we feel our body is good, we don't go to the hospital (P, 19)”.*

The availability of community sensitization is reported to be important, which would influence women to attend cervical cancer screening services.

*“If there is a community sensitization about the disease and significance of screening, I will be encouraged to undergo the screening test (P, 7)”.*

*“The challenge I have is I don't even know the signs and symptoms of the disease. If I receive education about the disease and screening test, I will go (P, 10)”*

Three participants stressed their desire to know their health status, which influenced them to participate in the screening test.

*“The desire to know my health status will influence me to go for screening (P, 23)”*

*“Because I know that prevention is better than cure, I can go to the screen for early detection and early treatment. After the screening, it helps to understand your health status, and this brings peace. Furthermore, I believe that there is no good life without the well-functioning of the reproductive organs. At the age of 30, I'm still young, I must do a regular check to remain healthy in my reproductive system, and even when I conceive, I'm confident that the pregnancy is safe (P, 2)”.*

Some other participants reported having free time, availability of screening services, free screening costs, and personal decisions may influence women to participate in CCS services

*“Once I get time, I will go for screening (P, 9)”*

*“If the screening service is available, I will go to screen (P, 15)”*

*If I get the money, I will go for screening (P, 16)”*

*“It is just a personal decision, when I decide, I will go to the screen. If I know where to get the service, I will go for screening (P, 18)”*

*“I think after I get focused and evaluate the effect of this disease, I will decide to attend the screening test (P, 4)”.*

*“Because I'm a woman and at risk of this disease, I am supposed to go to screen for cervical cancer to know my health status (P, 11)”.*

### Readiness for cervical cancer screening

Out of twenty-three participants, twenty-two participants demonstrated their readiness to undergo cervical cancer screening;

*“I'm confident I will go to screen for cervical cancer because checking health status is most important (P, 11)”.*

*“I'm confident to go for screening because I'm an adult woman with some understanding that cervical cancer can affect my reproductive organ, the organ I believe to be very important in my life. If I don't go for screening, this organ will be affected and will be removed from my body. Thereafter, I will not be called a woman because the reproductive organ is removed (P, 2)”.*

Participants demonstrated readiness to be screened, but they suggested that screening services to be available, cost-effective, and community education should be delivered.

*“If there is an opportunity for the availability for the test, I'm confident I will attend (P, 10)”*

*“Once there is the availability of the test service, I will be ready to attend for screening. Knowing the costs for cervical cancer screening, and whether the cost in public healthcare facilities differs from costs in private healthcare facilities, after knowing this, it will influence my readiness (P,13)”.*

*“If I receive the education about where the screening service is provided, how it is provided and no long waiting time, I'm sure to undergo the test (P, 8)”.*

### Cervical cancer screening practices

Only two participants shared their screening habits, including when they started attending screening services, and some information about screening intervals and age.

*“I screened once in 2019, I must screen again in 2022, which is the interval of 3 years. I'm committed to the schedule I have been given (P, 6)”.*

*“I did it once in 2019. I have not heard again if there is this opportunity. I wish I could have this opportunity to screen again. I don't remember if they told me to go back to the screen again after some time. I'm sure they gave me the form after screening; I will check if I'm supposed to go back again for screening. If the time is not due, I will ensure I get screened according to their schedule. I have the habit of 6-monthly check-ups of my health status regarding other diseases to know the status (P,12)”.*

## DISCUSSION

This study aimed to explore trends in cervical cancer screening. Participants in the present study had a mean age of 34.62 years, which is within the recommended age for cervical cancer screening, and it falls in the women's age group (20–40) reported at risk of cervical cancer ^28^. Most of the participants were self-employed in some small businesses to earn income, single, and had a primary school level of education. Only 2 (7.7%) had previously screened for cervical cancer by visual inspection with acetic acid (VIA).

### Awareness of cervical cancer and CCS

The study assessed the awareness of participants on cervical cancer and cervical cancer screening. All participants in this study were aware of cervical cancer and cervical cancer screening, but only 2 out of 26 participants had been screened for cervical cancer. The awareness is consistently reported in other previous studies, as 86% ^29^ and 70.8%.^30^ Some participants reported having heard information about CC and CCS from the ongoing national community awareness education program; however, a large number knew nothing about CC and CCS. This outcome brings uncertainty on how programs are being operated, whereas this was initially launched in 2014 ^9^. Furthermore, they reported having heard this information from different Media, especially radios and televisions, which is consistent with the previously conducted study in Ethiopia.^31^ Participants who have heard about cervical cancer and CCS have no details about cervical cancer and CCS, this is because they never pay attention or concentrate to understand the information they hear. Although the Media promotes awareness, the content delivered through this source might not be sufficient for recipients. This calls for strengthening the ongoing national awareness programs to come up with a new strategy to promote women's deeper understanding of cervical cancer and CCS. This is consistent with findings from the previous study recommended that the content and accuracy of messages delivered through Media have not yet been investigated. This requires the authorities to examine and innovate novel strategies.^6^

### Knowledge level of cervical cancer and CCS

The study aimed to investigate the knowledge of cervical cancer and CCS among participants. Only a few participants were able to identify that cervical cancer is a dangerous and fatal disease that occurs in the cervix and affects women's reproductive organs. Many women did not know the exact body part in a woman does cervical cancer occurs. Inadequate knowledge was found more on cervical cancer, causes, risk factors, signs and symptoms, prevention, and screening tests. The finding is consistent with the previous study reported that 4.0% of participants appeared to have a good level of knowledge of cervical cancer in risk factors, vulnerability, signs and symptoms, ways of prevention, and ways of screening.^32^

In the present study, even participants who had previously screened for cervical cancer were not able to demonstrate basic knowledge of cervical cancer. This might be due to poor performance of healthcare providers who are responsible for the screening department; they focus much on conducting the tests with little or no pre-screening education and post-screening education. No participant mentioned human Papillomavirus as the cause of cervical cancer. Although participants mentioned the risk factors of cervical cancer, they just guessed general factors to cause any type of cancer, but not specific to cervical cancer. They reported the use of chemical products as food or in bathing, putting themselves at risk. Participants were not sure about the signs and symptoms of cervical cancer, but just mentioned the problems that women often encounter in their daily lives, such as lower abdominal pain, abnormal vaginal discharge, and vaginal bleeding. Although one participant reported that avoiding multiple sexual partners and sexual intercourse at a young age could prevent cervical cancer, among them had no clear knowledge about the prevention measures. Participants were not familiar with CCS information and CCS guidelines, as they failed to identify the criteria for women to undergo CCS, which is supported by previous studies.^33^ Although most of the participants reported women aged 18 years old and above being eligible for screening, it is contrary to the recommended screening age of 21 years old and above. Participants self-reported that the lack of a community education program contributed to their poor knowledge. The inadequate knowledge among participants in this study might have been influenced by the participants’ level of education, as many of them 14 (53.8%) had a primary level of education. This is consistent with the previous data reported that a lower education level is associated with poor participation in screening services.^34^

### Hindrances to undergoing cervical cancer screening

Obstacles restricting women from participating in cervical cancer screening were identified. Having no signs or symptoms of cervical cancer was a leading hindrance to the participation of women in screening. This is supported by the previous finding from the study conducted in Uganda, which reported that experiencing signs and symptoms of CC triggered women to participate in CCS services.^35^ The second leading hindrance was the lack of community education programs in promoting community understanding of CC and CCS. This finding is supported by the previous study reported that women would have gone for the screening if they had received adequate information about the disease.^36^ Some participants mentioned screening costs as a hindrance to the participation of CCS; however, the cervical cancer screening services in Tanzania are delivered free of cost to all eligible women throughout the country.^37^ Having no free time to attend the tests emerged in the present study, which is similar to the previous finding showing that some women are busy taking care of their children, and others are working on their business, which makes it impossible for them to travel a distance to seek the screening services.^38^ This calls for the government efforts to keep on decentralizing screening services to the community level to reduce the distance to the healthcare facilities. Some respondents reported that the screening sites in Dar es Salaam are always overcrowded by women seeking screening services, causing long waiting times for the service. The workforce should be increased, and adequate infrastructure put in place for the convenience of women. Some participants had no reason why they had not gone to screening services; however, carelessness and poor health-seeking behavior were factors restraining their decision to undergo the tests. This is consistent with previous findings that poor health-seeking behavior makes a great contribution to women's poor participation in CCS services.^39^ The previous study reported that women with poor health-seeking behavior were 6.25 times more likely to have poor knowledge.^40^ In contrast with this previous study,^41^ women in the present study did not report fear of pelvic examination as a barrier to participating in CCS services.

### Attitude and belief toward cervical cancer screening

The attitude and health beliefs towards cervical cancer screening were explored. Twenty-four participants had never screened for cervical cancer but had positive attitudes and beliefs toward cervical screening. Since the women in this study were aware of cervical cancer and screening, this might be a reason for a positive attitude. It is supported by the previous study that reported that awareness has a good role in attitude formation.^42^ Even the cognitive learning theory reveals that having heard something from peers or the media influences someone to acquire an attitude.^43^ Even though several studies report that whenever people are knowledgeable, it is likely that their attitudes are positive,^44–46^ it is contrary to the current study, as women with inadequate knowledge of cervical cancer had a positive attitude. This might be because the study was conducted in an urban area where there might be multiple factors causing attitude change. They self-reported that the CCS test is good, and believed that women should undergo this kind of test because cervical cancer is dangerous and kills many women. The favorable attitude toward cervical cancer predicts the intention to undergo CCS.^31^

### Influences to undergo cervical cancer screening

Participants identified some factors that would lead them to go for screening, and among them said that after they get sick will go for screening. In the previous study, women reported that they only seek medical attention when they present signs and symptoms.^47^ This kind of wrong perception is influenced by inadequate knowledge about the disease and the significance of the CCS test. Community education about cervical cancer and CCS was mentioned as a factor that would influence women to attend CCS services. With few participants having undergone CCS, some participants mentioned the availability of screening services as their determinant for screening participation; however, the previous finding reported that in 2011, the government had launched 300 CCS sites across the country to ensure the availability and accessibility of screening services.^8^ This means that the screening services are available everywhere in the country; it is now an individual decision to seek the service. Furthermore, many screening sites are in every part of Dar es Salaam, the region where the government has implemented many CC prevention measures. Therefore, the availability of screening in Dar es Salaam doesn't seem like a hindrance. The desire to know their health status was self-reported by some participants; this is consistent with the previous findings showing that the urge to know whether their bodies are healthy or not was a determinant for women to seek screening services.^48^ Free CCS services were further mentioned as a factor identified by participants that would influence them to undergo CCS.

### Readiness for cervical cancer screening

The readiness to undergo screening among women was assessed, whereby the unscreened twenty-two out of twenty participants were ready to undergo CCS, which is consistent with previous findings that reported that 90% of women in Tanzania had demonstrated the readiness to screen for cervical cancer.^6^ This is further supported by a previous study that reported 88.9% were willing to screen for cervical cancer.^49^ Their readiness to screen might have been triggered by their high awareness of cervical cancer and screening, coupled with a positive attitude. This is supported by the previous study, which revealed that there is a significant difference in awareness and readiness.^50^ Moreover, the positive attitude was previously reported as a predictor of readiness to practice.^51^ Among those who were ready to screen, they suggested the availability of community education regarding the disease, free screening, and the availability of CCS services without long waiting times in the facilities. Whether participants had no idea, but the previous study reported that the screening services in Tanzania are provided at no cost.^52^ It is the responsibility of the government and the oncology department to strengthen the national awareness educational programs. The ongoing community awareness education program is good but not effective in influencing women to screen for cervical cancer; rather, it should be coupled with a knowledge educational program that will promote the understanding of the disease and encourage them to go for screening.

### Cervical cancer screening practices

The practice of women towards cervical cancer screening was investigated, whereby only two participants had previous screening tests. They had screened once, with 3-yearly screening intervals. One participant, after the first test, did not know whether she was required to screen again. Pre-screening education and post-screening education are not adequate to empower screened women to adhere to their scheduled appointments.

### Study limitations

The study relied on one population, which cannot confirm each identified concept. The study focused on in-depth interviews, while Focus Group Discussion (FGDs) would have yielded extensive findings. The study is limited as cultural norms as a screening hindrance are silent. Since some of the concepts were subjectively assessed rather than actual observation, it limits the generalizability of the study findings. Moreover, the purposive sampling of households is associated with the selection bias of participants that might have affected the findings. The study might have encountered a social desirability and recall bias aspect, especially in the question that demanded participants to describe their practices on cervical cancer screening. All of which might have affected the study conclusion. Additionally, the mean interview duration was, which might have affected the extensive exploring of issues and rapport building.

## CONCLUSION

Most women had a higher level of awareness because of the ongoing community awareness educational program, but a small proportion had screened for cervical cancer. To some extent, the ongoing community awareness educational programs have helped women to recognize how dangerous the disease is and promoted their positive belief but has not effectively influenced them to participate in cervical cancer screening tests. A lack of knowledge about the basic matters of cervical cancer and CCS has emerged in this study as the leading hindrance for women to screen for cervical cancer. There is a great demand for the government with other healthcare stakeholders, to reform and strengthen the community awareness educational program. Moreover, the cost and lack of free time of attending the facility for screening seem to hinder women from screening, calling on the government to introduce screening outreach services near people.
